# Detection of recurrence of HPV-driven oropharyngeal cancer by HPV cell-free DNA

**DOI:** 10.1038/s41598-026-56914-7

**Published:** 2026-06-13

**Authors:** Noemi M. Fricke, Vittoria Guarda, Daniela Höfler, Tim Waterboer, Charlotte S. Schouten, Kristian Ikenberg, Lea Schroeder, Fabian Rosing, Martina A. Broglie Daeppen

**Affiliations:** 1 Division of Infections and Cancer Epidemiology, German Cancer Research Center (DKFZ), Heidelberg, Germany; 2 Faculty of Biosciences, Heidelberg University, Heidelberg, Germany; 3 Department of Otorhinolaryngology, Head and Neck Surgery, University Hospital of Zurich, Zurich, Switzerland; 4 Department of Pathology and Molecular Pathology, University Hospital of Zurich, Zurich, Switzerland

**Keywords:** Biomarkers, Cancer, Oncology

## Abstract

The incidence of oropharyngeal cancer (OPC) driven by human papillomavirus (HPV) is increasing in high-income countries. Recurrent tumors remain a major cause of HPV-OPC mortality, yet reliable biomarkers for post-treatment monitoring are lacking. This study evaluated HPV cell-free DNA (cfDNA) from blood plasma as a monitoring biomarker for the detection of HPV-OPC recurrence. Blood plasma from 59 OPC patients was collected at diagnosis and during follow-up. HPV cfDNA was quantified using a multiplex digital PCR assay targeting HPV16 and seven other high-risk types. Tumor HPV status was confirmed by p16^INK4A^ immunohistochemistry, HPV DNA PCR, and E6 serology. At diagnosis, HPV cfDNA was detected with 95% sensitivity (36/38; 95% CI 82–99%) and 95% specificity (19/20; 95% CI 75–100%). For 24 HPV-OPC patients, follow-up samples were available (median follow-up 1.5 years), with four patients experiencing recurrence or persistence of HPV-OPC. In two patients, HPV cfDNA detection after initial clearance preceded clinical recurrence by 3 and 8 months, respectively. The third patient experienced recurrence 7 months after collection of the last blood sample, which was HPV cfDNA-negative. The fourth patient had persistent disease without HPV cfDNA detection. HPV cfDNA positivity after therapy had a positive predictive value of 75% for HPV-OPC recurrence within one year on a per-test basis. HPV cfDNA has been shown to have great potential as a minimally invasive monitoring biomarker for HPV-OPC surveillance. Monitoring HPV cfDNA levels may identify patients at risk of recurrence early, thereby potentially facilitating timely intervention. Studies with more participants are needed to establish a solid evidence base for surveillance protocols that incorporate HPV cfDNA.

**Supplementary Information:**

The online version contains supplementary material available at 10.1038/s41598-026-56914-7.

## Introduction

The growing burden of oropharyngeal cancer (OPC) is largely attributable to the increasing incidence of cases driven by infections with high-risk human papillomavirus (HPV) types, particularly HPV16^1^. HPV-OPC incidence varies depending on the geographical region, with the highest incidence found in regions with a high human development index, such as North America and Europe, and its increase is expected to continue for the next 20–30 years^2–4^. In the US and UK, OPC has surpassed cervical cancer as the most common HPV-associated malignancy in 2012 and 2016, respectively^2^.

As the HPV status has implications for OPC staging, treatment response and prognosis, it is routinely determined in tissue biopsies^5^. p16^INK4A^ (p16) positivity in immunohistochemical staining (IHC) is accepted as surrogate marker for HPV-driven carcinogenesis in the oropharynx^6^, although additional testing for HPV nucleic acids in tumor tissue is recommended^7–9^. Alternatively, serum antibodies directed against HPV early proteins, particularly E6, have been described as a highly sensitive and specific diagnostic marker for HPV-OPC^10,11^.

Despite the overall favorable prognosis of HPV-OPC, recurrence occurs in 15–25% of patients within 3 years of treatment completion^12^. Recurrent disease is associated with worse patient outcomes and increased mortality. A reliable surveillance strategy for OPC patients could increase the chances for timely intervention and thereby improved outcomes. However, early identification of the patient subset that will experience disease recurrence remains challenging, as no biomarkers have been established so far.

HPV cell-free DNA (cfDNA) isolated from blood has emerged as a promising minimally invasive biomarker for treatment response and surveillance of HPV-OPC in multiple studies^13–17^. cfDNA may be released into the bloodstream by dying tumor cells and is usually fragmented and low in concentration^18^. Therefore, the detection of cfDNA requires highly sensitive techniques, such as digital PCR (dPCR). In dPCR, the sample is analyzed in thousands of individual partitions, allowing for absolute target quantification and higher sensitivity compared to conventional PCR methods^19^.

Here, we further evaluated the performance of HPV cfDNA as a blood-based biomarker for the early detection of OPC recurrence, utilizing an adaptation of a recently published dPCR assay^17^ for accurate quantification of cfDNA from eight high-risk HPV types.

## Methods

### Patient cohort and definition of tumor HPV status

The study cohort included patients with newly diagnosed OPC or cancer of unknown primary (CUP) within the framework of a prospective, observational biomarker study, involving patients with histologically confirmed head and neck squamous cell carcinoma (HNSCC). The study was approved by the Ethics Committee of the Canton of Zurich (No. 2018–02126). Research was performed in accordance with relevant institutional guidelines, regulations and the Declaration of Helsinki. Patients of both sexes were enrolled from March 2020 to May 2023 at the Department of Otorhinolaryngology, Head and Neck Surgery, University Hospital of Zurich, Switzerland. Written informed consent was obtained from all patients prior to study entry. Patient clinical and sociodemographic characteristics (including age, sex, performance status and disease-specific risk factors) and follow-up data were obtained from questionnaires and clinical charts.

Stage was determined according to the 8th edition of the Union for International Cancer Control (UICC)/American Joint Committee on Cancer (AJCC) TNM system. The patient’s performance status was graded on a scale from 0 to 5 according to the Eastern Cooperative Oncology Group (ECOG)^20^. Treatment was defined by the institutional tumor board according to international guidelines. The study cohort included patients treated with definitive surgery alone, definitive radiation therapy (RT), definitive chemoradiation (CRT) and surgery with adjuvant RT or CRT.

After enrollment, i.e., at time of diagnosis, 2 × 10 ml blood in EDTA and 10 ml blood in serum separator tubes were collected and clinical and radiological data were obtained. If radiotherapy was performed, further visits took place at 1 and 4 weeks after the start of radiotherapy, where respectively 10 ml blood in EDTA and in serum separator tubes were collected. Furthermore, follow-up visits were scheduled at 6–8 weeks, 3, 6, 9, 12, 15, 18 months after the end of treatment and surveillance data were collected. EDTA blood was centrifuged for 15 min at 3400 rpm at 4 °C and plasma was stored at -80 °C. In addition, if surgery was performed, a follow-up visit was scheduled 1–2 weeks after resection. Deviations from the time intervals specified in the protocol were due to restrictions as well as to patients’ reluctance to undergo additional measures in the hospital during the COVID-19 pandemic.

Tumor specimens routinely obtained during diagnostic panendoscopy or from surgical tumor resection (i.e. for CUP) were assessed at the Institute of Pathology and Molecular Pathology (University Hospital of Zurich, Switzerland) for HPV status using p16 IHC. p16 IHC positivity was confirmed by HPV-DNA testing using consensus primer PCR^21,22^ with subsequent sequencing. Serum samples at time of diagnosis were analyzed for serum antibodies against E6 and E7 antigens of HPV types 16, 18, 31, 33, 35, 45, 52 and 58, and additionally E1 and E2 antigens of HPV16 by HPV multiplex serology (DKFZ, Heidelberg, Germany) as previously described^22^. A serum sample was considered seropositive for a given HPV type if it was seropositive for antibodies against the E6 antigen of that HPV type. A serum sample was also considered HPV16-seropositive if it was seropositive for antibodies against all three of HPV16 E7, E1 and E2^10^. Multiple infections in HPV-OPC are rare, however there is some cross-reactivity between antibodies against closely related HPV types. Therefore, if a sample was seropositive for multiple HPV types, the HPV type with the highest E6 median fluorescence intensity (MFI) value was considered the causative HPV type, as cross-reactive signals are expected to be lower than the signal of the tumor-driving HPV type. A tumor was classified as HPV-driven if at least 2 out of 3 biomarkers (p16, HPV DNA and HPV serology) were positive.

### cfDNA isolation, HPV cfDNA quantification and data analysis

Isolation and quantification of HPV cfDNA was performed at DKFZ (Heidelberg, Germany). Plasma samples were shipped to DKFZ from Zurich on dry ice. Available plasma volume ranged from 0.5 to 4 ml (median 2.4 ml). cfDNA was isolated from plasma using the automated MagNA Pure 96 system (Roche Diagnostics, Mannheim, Germany) with the DNA and Viral NA Large Volume Kit (Roche Diagnostics, Mannheim, Germany) according to the manufacturer’s instructions. The cfNA ss 2000 or cfNA ss 4000 purification protocol was used depending on the available plasma volume, which was adjusted to a final volume of 2 or 4 ml with PBS if necessary. PBS was used as the negative control for extraction, while a sample containing plasmids of all target sequences was used as extraction positive control. cfDNA was eluted in 50 µl elution buffer.

HPV cfDNA was quantified using a previously described multiplex dPCR assay detecting HPV16, 18, 33, 45, 52, 58 as well as human beta-globin (BG) as a reference gene to control for sufficient input material^17^. Experimenters were blinded to the patient’s tumor HPV status. Samples were tested in duplicates with 20 µl cfDNA per run using QIAcuity 26k 24-well nanoplates on the QIAcuity One (QIAGEN, Hilden, Germany), with a median number of 25,400 valid partitions analyzed per well. As an additional channel became available on QIAcuity by a software update during the study period, the assay was expanded to also cover HPV31 and HPV35 (Supplementary Table 1). The expanded assay was conducted using the QIAcuity High Multiplex Probe PCR Kit. Samples from all OPC cases at diagnosis and follow-up samples from the HPV35-driven OPC case were re-tested using the expanded panel using a reduced input volume of 5 µl per reaction due to sample volume limitations (Supplementary Fig. 1). The dPCR cycling conditions are listed in Supplementary Table 2. Water was used as PCR negative control, while a sample containing plasmids carrying all target sequences was used as PCR positive control. Samples were considered positive for human BG if a minimum of 20 copies was detected in each duplicate. All plasma samples were positive for human BG and therefore valid. All negative controls were negative for BG. In case a sample had HPV cfDNA results that contradicted the results of other samples from the same patient, SNP profiling was performed to verify whether the samples were indeed from the same patient^23^. SNP profiling ruled out the possibility of a sample mix-up in all cases. Samples from one HPV69-positive case were excluded from dPCR analysis as HPV69 is not a generally accepted high-risk HPV type and therefore not included in our HPV cfDNA target panel. Additionally, insufficient data was available to verify that HPV was transcriptionally active in this patient. The HPV69-positive patient was retained for the sociodemographic and clinical comparison between HPV-positive and HPV-negative groups.

Data was processed and analyzed as previously described^17^, using the QIAcuity Software Suite (version 2.5.0.1), R (version 4.3.3) and RStudio (RRID: SCR_000432, version 2023.12.1.402). Fluorescence thresholds were calculated for each channel and each dPCR plate individually as the midpoint between the median RFU values of positive and negative partitions of a positive control. The same threshold was applied to all wells on the plate. A sample was considered as HPV cfDNA positive if there was at least one positive partition in each of the duplicates with a mean absolute z-score < 2. If a positive partition was present in only one of the duplicates, the result was labeled as inconclusive and treated as a negative, as specificity was prioritized (Supplementary Table 3). HPV copy numbers were normalized to the corresponding plasma volume used for cfDNA isolation. For sensitivity and specificity, the Clopper-Pearson confidence interval (CI) with α = 0.05 was calculated. The main outcomes investigated were sensitivity and specificity in discerning HPV-OPC and HPV-negative OPC, and the positive (PPV) and negative predictive values (NPV) for HPV-OPC recurrence or persistence after treatment (Supplementary Tables 3 and 4). PPV and NPV were calculated on a per-test basis for OPC recurrence or persistence within 12 months of the respective test result, using biopsy-proven clinical recurrence/persistence as the reference standard. Given the absence of a perfect gold standard, our reported metrics reflect agreement with clinical assessment and correspond to positive percent agreement (PPA) for sensitivity, negative percent agreement (NPA) for specificity, as well as positive and negative concordance for PPV and NPV, respectively^24^. For readability and consistency with similar studies, conventional terminology is used here.

## Results

### Sociodemographic and clinical differences based on tumor HPV status

The study cohort comprised 58 OPC patients and 1 CUP patient. Patients were grouped according to tumor HPV status, with 39 HPV-driven and 20 HPV-negative patients. The majority of HPV-OPC was solely positive for HPV16 (*n* = 33, 84.6%), while two tumors were HPV16/HPV33 double positive. Three OPC cases were positive for HPV33, HPV35 and HPV58, respectively. One patient was positive for HPV69, which is not a recognized high-risk HPV type. This patient was excluded in the subsequent dPCR analysis.

Patients with HPV-negative OPC had a higher prevalence of alcohol consumption (55% vs. 13%) and smoking (90% vs. 49%) compared to patients with HPV-OPC (Table 1). More than 90% of patients with HPV-negative OPC presented with advanced disease (stage III or IV), in comparison to 26% of patients with HPV-OPC.

**Table 1:** Baseline characteristics in OPC/CUP patients by tumor HPV status.

Characteristic	HPV-driven *N* = 39^1^	HPV-negative *N* = 20^1^
Age (median. IQR)	68 (61, 72)	69 (65, 77)
Age (mean ± SD)	68 ± 8.7	70 ± 8.5
Sex		
Male	25 (64%)	14 (70%)
Female	14 (36%)	6 (30%)
Tobacco (> 10 pack-years)	19 (49%)	18 (90%)
Alcohol consumption (≥ 3 Units/day)	5 (13%)	11 (55%)
ECOG performance status		
0	27 (87%)	9 (56%)
1	4 (13%)	4 (25%)
2	0 (0%)	2 (13%)
3	0 (0%)	1 (6.3%)
Unknown	8	4
Tumor site		
Oropharynx	38 (97%)	20 (100%)
CUP	1 (2.6%)	0 (0%)
Subsite		
Tonsil	17 (44%)	9 (45%)
Base of tongue	13 (33%)	4 (20%)
Lateral or posterior wall of oropharynx	3 (7.7%)	4 (20%)
Tumor spanning multiple anatomic sites	3 (7.7%)	2 (10%)
Other	3 (7.7%)	1 (5.0%)
Treatment		
No therapy	0 (0%)	1 (5.0%)
Surgery	6 (15%)	1 (5.0%)
Radiotherapy (RT)	7 (18%)	9 (45%)
Radiochemotherapy (RCT)	24 (62%)	8 (40%)
Surgery + adjuvant RT/RCT	2 (5.1%)	1 (5.0%)
Stage (TNM8)		
I	21 (54%)	0 (0%)
II	8 (21%)	1 (5.0%)
III	10 (26%)	5 (25%)
IV	0 (0%)	14 (70%)
T		
0	1 (2.6%)	0 (0%)
1	10 (26%)	3 (15%)
2	15 (38%)	5 (25%)
3	5 (13%)	2 (10%)
4	8 (21%)	10 (50%)
N		
0	4 (10%)	6 (30%)
1	24 (62%)	2 (10%)
2	11 (28%)	7 (35%)
3	0 (0%)	5 (25%)
M		
0	39 (100%)	20 (100%)
p16 IHC		
Negative	0 (0%)	17 (85%)
Positive	39 (100%)	1 (5.0%)
Inconclusive	0 (0%)	2 (10%)
HPV DNA		
Negative	2 (5.3%)	8 (100%)
Positive	36 (95%)	0 (0%)
Unknown	1	12
HPV type		
HPV16	34 (87%)	
HPV33	2 (5.1%)	
HPV35	1 (2.6%)	
HPV58	1 (2.6%)	
HPV69	1 (2.6%)	
Second primary head and neck cancer	0 (0%)	2 (11%)
Unknown	1	1
One or more additional oncological diagnoses	7 (18%)	8 (42%)
Unknown	0	1
Thereof:		
Bladder cancer	3	1
Prostate cancer	2	1
Prostate cancer and myeloproliferative neoplasm	1	0
Non-melanoma skin cancer	1	2
Lung cancer	0	2
Lung cancer and melanoma	0	1
Breast cancer	0	1

^1^n (%).

Values represent either the total number and percentage of patients (categorical variables), the median value with interquartile range (IQR) or the mean value with standard deviation (SD) (continuous variables).

HPV-negative patients had an overall higher ECOG status, and thus an overall worse performance status.

The HPV-driven and HPV-negative groups did not differ in age (68 vs. 69), gender (64% vs. 70% male) nor tumor location, with the most frequent subsite in both groups being the tonsils (44% and 45%, respectively).

### Clinical follow-up and outcomes

OPC patients were under clinical follow-up for a median of 1.2 years (range 20 days – 2.9 years) and outcomes were recorded. Seven patients died during the follow-up period, all of whom had HPV-negative OPC (35%, 95% CI 15–59%) with advanced disease (clinical stage III or IV) (Table 2). Deaths occurred at a median of 7 months (range 20 days – 1.9 years) after diagnosis. Similarly, recurrent disease was more common in patients with HPV-negative OPC (*n* = 6; 43%, 95% CI 18–71%) compared to those with HPV-OPC (*n* = 3; 8.8%, 95% CI 2–24%) (Table 2). OPC recurrence was diagnosed at a median of 15 months (range 6–20 months) after diagnosis among patients with available timing information (*n* = 8).

**Fig. 1:**
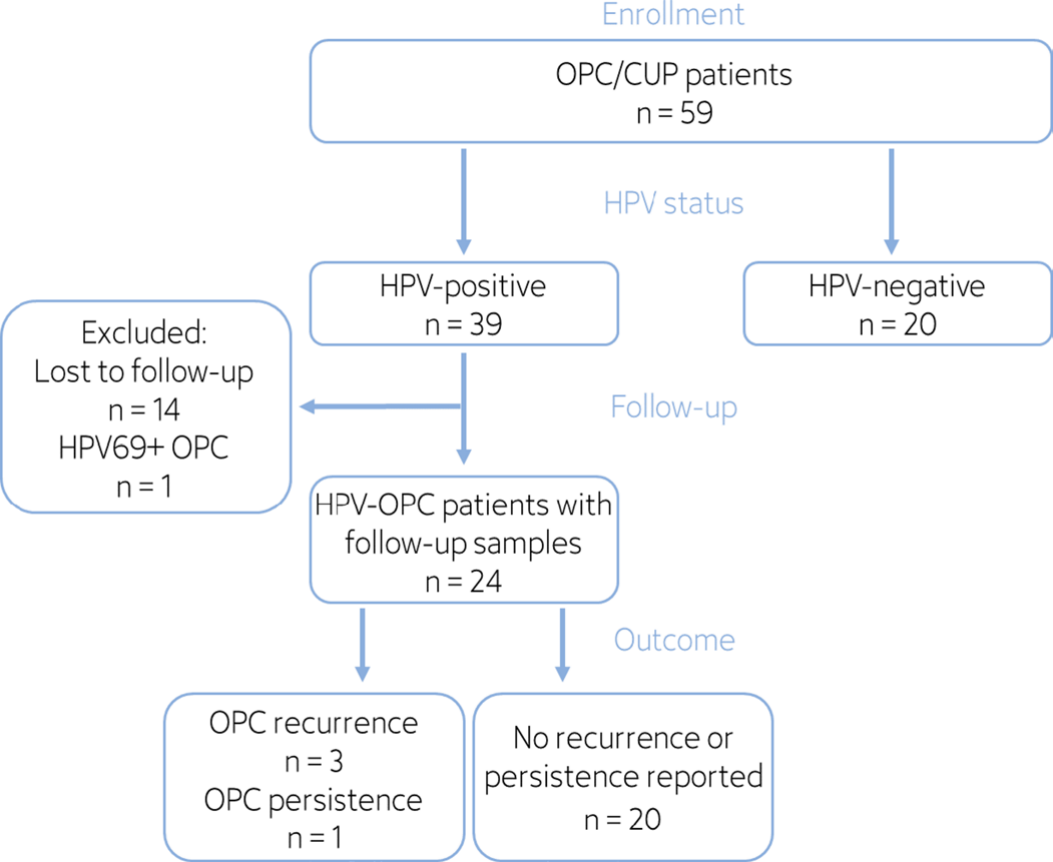
Flowchart of study participants.

All OPC patients provided blood samples at diagnosis, while follow-up samples were analyzed for 24 of 39 HPV-OPC patients (Fig. 1). Fourteen HPV-OPC patients did not provide follow-up samples: Nine only gave consent to analyze samples at diagnosis due to the pandemic, two withdrew consent to collection of follow-up samples, and three were lost to follow-up because radiotherapy was performed as an outpatient procedure at a different facility. One additional HPV-OPC patient was excluded from further analyses due to not testing positive for a high-risk HPV type included in the test panel.

**Table 2:** Clinical outcomes in OPC/CUP patients by tumor HPV status.

Outcome	HPV-driven *N* = 39^1^	HPV-negative *N* = 20^1^
Persistent disease	1 (2.6% [0–13%])	2 (13% [2–38%])
Unknown	0	4
Recurrent disease	3 (8.8% [2–24%])	6 (43% [18–71%])
Unknown	5	6
Death	0 (0% [0–9%])	7 (35% [15–59%])

^1^n (%).

Square brackets indicate the 95% Clopper-Pearson confidence interval.

### Concordance of HPV cfDNA in blood and HPV status in tissue at time of diagnosis

The presence and quantity of HPV cfDNA at time of diagnosis in the blood plasma of patients with HPV-OPC (*n* = 38) and HPV-negative OPC (*n* = 20) were investigated using a multiplex dPCR assay detecting the eight high-risk HPV types HPV16, 18, 31, 33, 35, 45, 52 and 58. All included plasma samples contained sufficient human beta-globin with a median of 2,190 copies/ml (range 548–685,777 copies/ml).

**Fig. 2:**
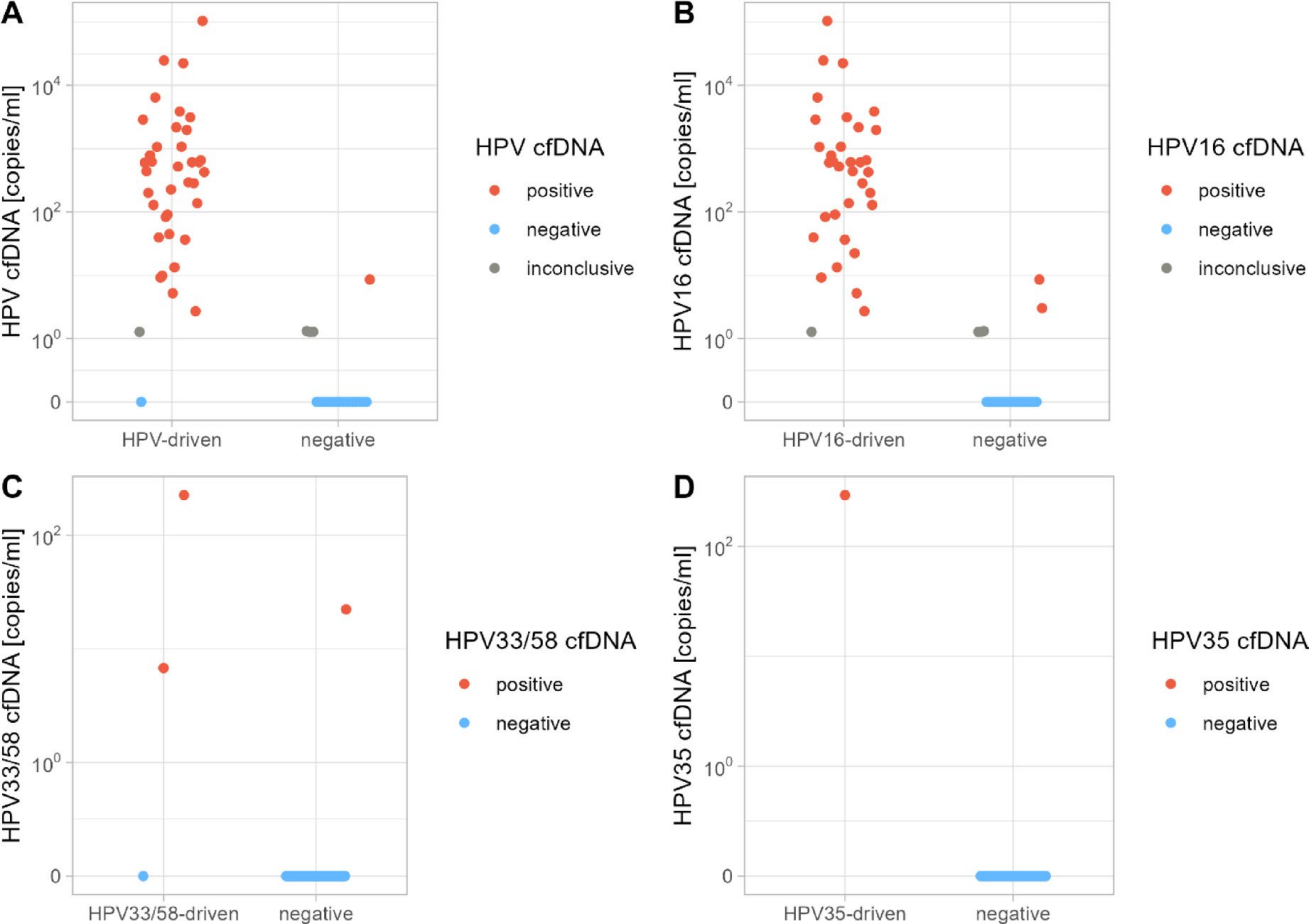
HPV cfDNA levels in blood plasma of 58 patients at time of diagnosis in relation to tumor HPV status. (**A**) Sum of HPV copies across all HPV types, and copies of (**B**) HPV16, (**C**) HPV33/58 and (**D**) HPV35 detected per sample. The HPV cfDNA status determined by dPCR was compared to the tumor HPV status. Mean copy numbers for HPV16 and HPV33/35/58 from two dPCR replicates per ml plasma volume are shown. Samples that were reproducibly tested positive or negative across both duplicates were classified as positive (red) or negative (blue), respectively, while samples tested positive in only one replicate were labeled as inconclusive (grey). Values of 0 were replaced with a value of 0.1 (here labeled as 0) to allow the display on a logarithmic scale. 58 out of 59 study participants are shown here, since samples from one HPV69-positive patient were excluded from dPCR analysis.

HPV cfDNA was present in 36 of 38 HPV-OPC patients at time of diagnosis, indicating a sensitivity of 95% (95% CI 82–99%, Fig. 2A). HPV cfDNA was not detected in two patients, one with HPV16-driven OPC and one with HPV33-driven OPC. Both patients had stage I OPC with minor lymph node involvement (N1).

Conversely, HPV cfDNA was not detected in 19 of 20 patients with HPV-negative OPC, resulting in a specificity of 95% (95% CI 75–100%). The single HPV cfDNA false positive patient had 8.5 copies/ml HPV16 cfDNA but was negative for p16 IHC and HPV16 E6 antibodies.

HPV cfDNA concentration in patients with HPV-OPC who tested positive for HPV cfDNA at time of diagnosis ranged from 3 to 104,009 copies/ml (median 560 copies/ml) for HPV16 and from 7 to 226 copies/ml (median 26 copies/ml) for HPV33, HPV35 or HPV58. A higher HPV cfDNA concentration was not statistically significantly correlated with any tumor parameters, although a positive trend for clinical, tumor and nodal stage was observed.

HPV genotypes identified by the dPCR assay matched the tumor HPV status in all cases. HPV-type specific sensitivity was 97% for HPV16 (33/34, 95% CI 85–100%), 50% for HPV33 (1/2, 95% CI 9–99%), 100% for HPV58 (1/1, 95% CI 0.025–100%) and 100% for HPV35 (1/1, 95% CI 0.025–100%) (Fig. 2B–D). Due to limited multiplexing capacity, only four channels were available to test for the eight HPV types included, and channel-wise specificity was 92% for HPV16 (22/24, 95% CI 73–100%), 98% for HPV33/52/58 (64/65, 95% CI 92–100%), 100% for HPV31/35 (67/67, 95% CI 95–100%) and 100% for HPV18/45 (68/68, 95% CI 95–100%).

### Monitoring HPV cfDNA levels supports early diagnosis of recurrence

Twenty-four HPV-OPC patients who were positive for HPV cfDNA at baseline were followed up for a median of 1.5 years (range 8.4 months – 2.4 years) after diagnosis, and data on the course of disease and final outcomes of the patients were collected. Ninety-one plasma samples from a median of 3 follow-up visits per patient (range 1–8) were collected after diagnosis and tested for HPV cfDNA. The first follow-up sample was taken at a median of 41.5 days (range 14–273 days) after diagnosis. Therapy started at a median of 21 days (range 1–37 days) after diagnosis and lasted for a median of 70 days (range 14–190 days) across all treatment modalities.

**Fig. 3:**
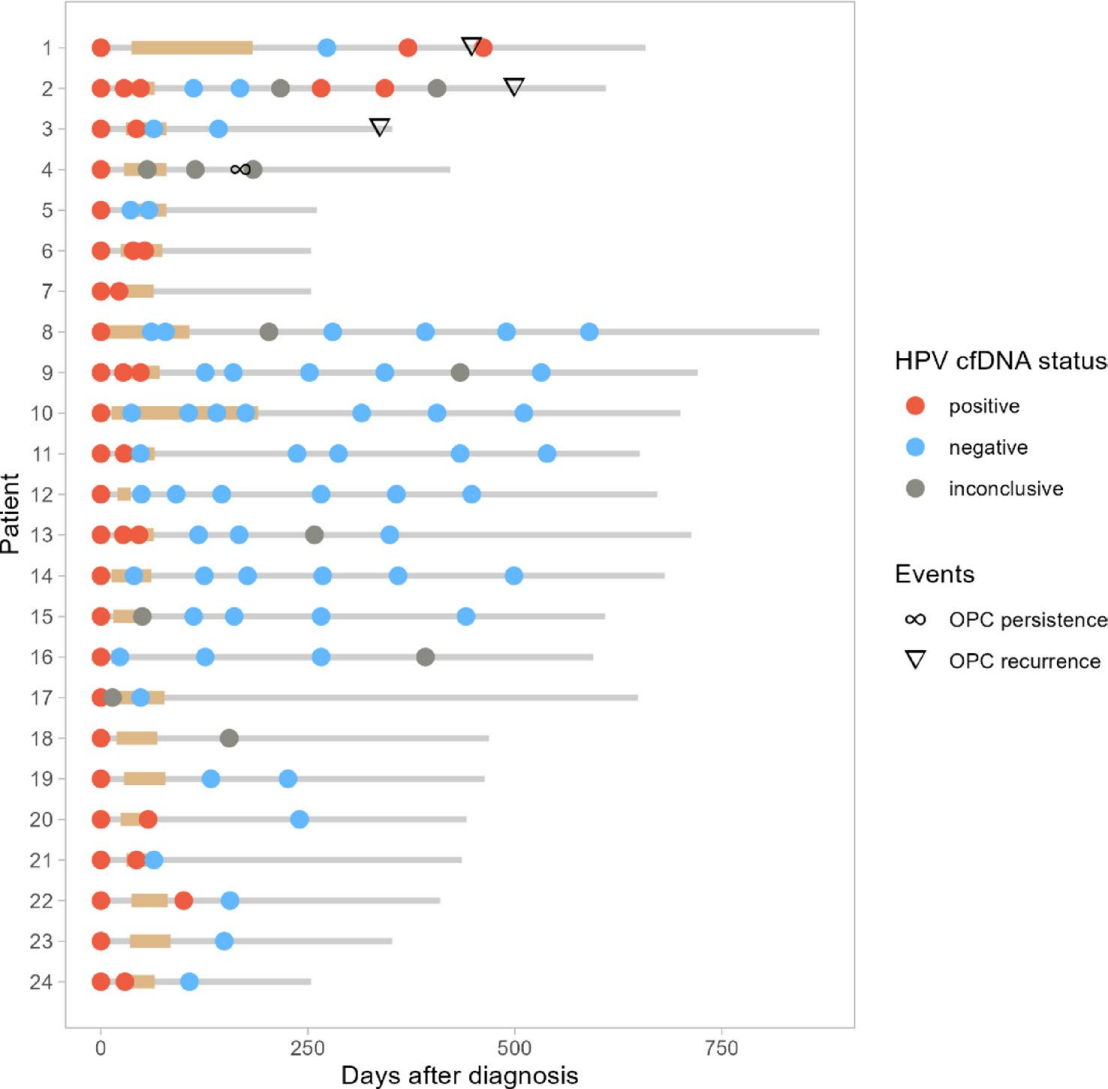
HPV cfDNA status in 24 HPV-OPC patients during the follow-up period. HPV cfDNA status for any HPV type determined by dPCR across all patient plasma samples over the course of disease. Samples that were tested positive or negative across both duplicates for any HPV type were classified as positive (red) or negative (blue), respectively. Samples that tested positive in only one replicate for any HPV type were labeled as inconclusive (grey), even if they were negative for the tumor-driving HPV type (e.g. for patient 4). Grey lines indicate the clinical follow-up period. Brown bars mark the therapy period. Patient outcomes (persistence, recurrence) are marked at the corresponding date on the timeline.

**Fig. 4:**
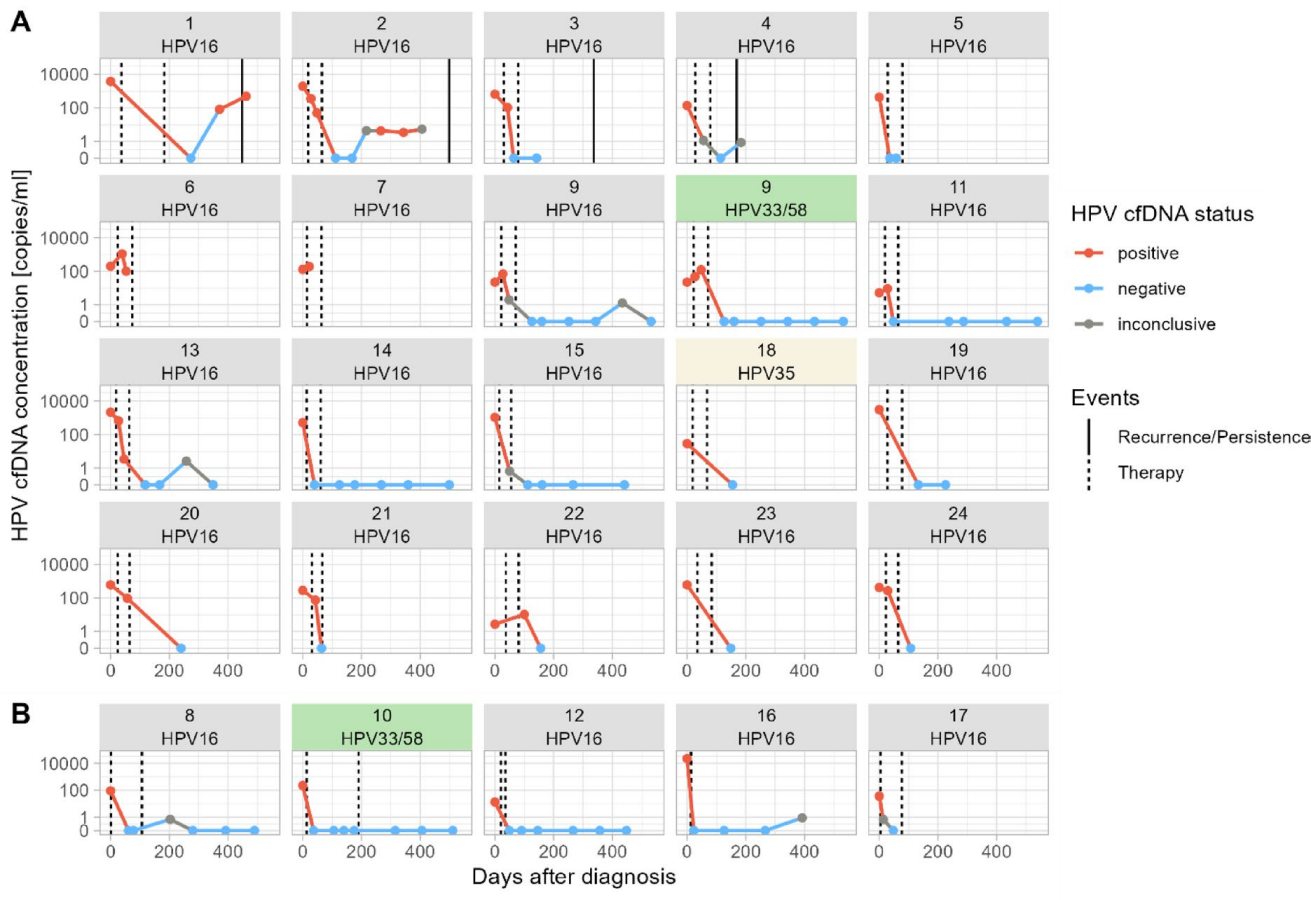
HPV cfDNA concentrations in 24 HPV-OPC patients during and after treatment. HPV cfDNA status for the respective indicated HPV type determined by dPCR across all patient plasma samples over the course of disease. For each subplot, a patient ID and the HPV type are indicated (HPV16: grey, HPV33/58: green, HPV35: yellow). Mean copy number of HPV16, HPV33/58 or HPV35 based on dPCR duplicates per ml plasma volume is shown. Samples that were tested positive or negative across both duplicates for the indicated HPV type were classified as positive (red) or negative (blue), respectively, while samples tested positive in only one replicate were labeled as inconclusive (grey). Therapy period (dashed lines) and time of disease recurrence or persistence (solid lines) are marked. Values of 0 were set to 0.1 (here labeled as 0) to allow display on a logarithmic scale. (**A**) Patients that did not receive surgery (RT/CRT only). (**B**) Patients that received surgery (with or without adjuvant RT/CRT).

Four patients within the HPV-OPC group experienced OPC recurrence or persistence during the follow-up period (Figs. 3 and 4). One patient (patient 1) presented with local recurrence at 15 months after diagnosis, while another patient (patient 2) was diagnosed with pulmonary metastases approximately 17 months after diagnosis. Both patients had a positive HPV cfDNA status at time of diagnosis (3853 and 1969 copies/ml, respectively), which were cleared upon completion of CRT. HPV cfDNA levels increased again prior to the clinical diagnosis of recurrence in both patients. HPV16 cfDNA concentration of patient 1 increased to 82 copies/ml three months before recurrence and further to 494 copies/ml two weeks after recurrence had been diagnosed. In patient 2, a positive HPV cfDNA status with 4 copies/ml was detected again seven months after end of therapy, and remained at this concentration for another three months. The first plasma samples consistently positive for HPV cfDNA post-treatment were collected approximately 3 and 8 months prior to the clinical diagnosis of recurrence in patient 1 and 2, respectively. The third patient (patient 3) presented with locoregional recurrence 7 months after collection of the last blood sample, which had negative HPV cfDNA status. One patient (patient 4) had persistent disease at end of therapy confirmed by imaging and biopsy, but their HPV cfDNA status remained negative after treatment.

Out of 24 patients with positive HPV cfDNA status at diagnosis and any available follow-up blood samples, 23 were HPV cfDNA-negative after treatment completion (Figs. 3 and 4). One patient (patient 22) had one positive HPV cfDNA result (10.4 copies/ml) shortly after end of therapy, despite not suffering a recurrence during the follow-up period, and further follow-up samples were negative. After completion of treatment this patient still had residual tumor mass, but a biopsy revealed no viable tumor cells. The other 19 patients had neither positive HPV cfDNA test results post-treatment nor recurrent or persistent disease reported.

To evaluate test performance in post-treatment surveillance, 59 HPV cfDNA test results from 24 HPV-OPC patients with follow-up were evaluated on a per-test basis. The 59 corresponding samples were taken after end of treatment and prior to a potential recurrence or persistence. If disease recurrence or persistence was diagnosed within 12 months of a positive HPV cfDNA result, the test was considered true positive. Conversely, if no recurrence or persistence was diagnosed within 12 months of a negative HPV cfDNA test result, the result was considered true negative. HPV cfDNA detection after completion of therapy had a positive predictive value (PPV) of 75% (3/4, 95% CI 19–99%) for recurrence of HPV-OPC within one year. Specificity was 98% (49/50, 95% CI 89–100%), as one test result during follow-up was false positive. The negative predictive value (NPV) for recurrence within one year was 89% (49/55, 95% CI 78–96%) and sensitivity was 33% (3/9, 95% CI 7–70%), both due to six false negative or inconclusive results across patients with OPC recurrence or persistence. Patient 1 and 4 each also had one sample collected shortly after their respective OPC recurrence or persistence, with a positive HPV cfDNA test result for patient 1 and an inconclusive HPV cfDNA test result for patient 4.

HPV cfDNA kinetics were compared between patients treated with RT or CRT (Fig. 4A) and those treated by surgery (with or without adjuvant treatment, Fig. 4B). As HPV cfDNA levels change rapidly after initiation of treatment, only patients with samples positive for HPV cfDNA at diagnosis and available follow-up samples within the first 4 months after diagnosis were included in this comparison (*n* = 20). Patients treated surgically (*n* = 5) had cleared their HPV cfDNA at the first follow-up sample after a mean of 37 days (range 14–61 days). Patients treated with RT or CRT alone (*n* = 15) had on average one positive sample after start of treatment (range 0–4), with the last positive sample at a mean of 34 days (range 0–100 days) after diagnosis. The first negative test result for HPV cfDNA occurred at a mean of 68 days (range 36–118 days) after diagnosis. Among patients treated with RT or CRT alone, a gradual decline of HPV cfDNA concentration was observed in some cases (Fig. 4A, patients 2 and 13). In two patients HPV cfDNA levels initially increased after start of treatment (Fig. 4A, patients 6 and 9), but their HPV cfDNA was eventually cleared.

## Discussion

There is a clinical need for reliable monitoring biomarkers of HPV-OPC recurrence. HPV cfDNA is a promising blood-based candidate, but its detection requires highly sensitive methods. In our cohort, HPV cfDNA had a PPV of 75% after completion of therapy for detecting recurrence of HPV-OPC, and was detectable three to eight months before recurrence was diagnosed based on imaging. With an NPV of 89%, post-treatment testing for HPV cfDNA might also provide reassurance to patients and reduce surveillance burden. Our findings are consistent with previous studies supporting HPV cfDNA as biomarker for OPC recurrence, with a similar reported lead time, albeit higher PPV^15,17,25^. For a potential role as a prognostic biomarker at diagnosis, our dPCR assay demonstrated high sensitivity (95%) and spcificity (95%) for the detection of HPV cfDNA from blood plasma of OPC patients at time of diagnosis, as HPV-OPC has a far better prognosis than HPV-negative OPC.

At diagnosis, there was one false positive result for HPV cfDNA in an HPV-negative tumor. As the tumor was p16-negative, it had not been tested for HPV DNA. Due to the lack of HPV16 E6 antibodies in this patient, one possible explanation could be that the tumor contained transcriptionally inactive HPV DNA. During post-treatment monitoring, there was a false negative result for one HPV-OPC patient with local treatment failure, even though this patient was positive for HPV cfDNA at diagnosis. It is possible that the remaining tumor after RT had too little contact to blood vessels or the lymphatic system to shed detectable amounts of HPV cfDNA. The HPV type-specific accuracy of HPV cfDNA detection was lower than expected, mainly due to two cases in which both HPV16 and HPV33, or HPV16 and HPV58 DNA were detected in the plasma sample. In both of these cases, tumor HPV DNA results were inconclusive. Both cases had antibodies against both HPV types detected in cfDNA. This may indicate that neither the tumor HPV DNA test nor the algorithm for interpreting HPV multiplex serology results were able to precisely detect presence of multiple HPV types in one tumor.

In one patient, 4 copies/ml of HPV cfDNA were detected before disease recurrence, again underlining the need for highly sensitive detection methods. When comparing patients treated with or without surgical resection of the tumor there is an apparent trend of quicker HPV cfDNA clearance among patients treated with surgery, but the cohort was too small to draw definitive conclusions. However, these findings align well with other published studies^26,27^.

All HPV-OPC patients who experienced disease recurrence or persistence were smokers with more than 30 py, which is in line with studies reporting a higher risk of recurrence for patients with HPV-OPC who consume tobacco^4,28^. Further commonly known trends were confirmed, such as a higher prevalence of alcohol consumption, smoking, disease recurrence and death in the HPV-negative group. Still, half of the HPV-OPC patients were also smokers, indicating that HPV-OPC also arises in populations with traditional risk factors, as has been described before^29^.

Other frequent observations in the literature were not found in this study. For instance, HPV-OPC patients usually present with higher nodal stage compared to HPV-negative patients^30,31^. Here, the nodal stage was generally higher for patients with HPV-negative OPC, but fewer HPV-OPC patients had no lymph node involvement at all. The correlation between HPV cfDNA concentration and disease stage was not statistically significant, even though a correlation between HPV cfDNA levels and nodal or clinical stage, but not tumor stage, has been described^32^.

There were several limitations of this study: With 59 patients, the study cohort was relatively small. This was exacerbated by the unavailability of follow-up samples for 14 out of 39 patients with HPV-OPC. This dropout, partly due to the COVID-19 pandemic, was unlikely to be random and may have introduced selection bias. As the clinical outcomes of those lost to follow-up are unclear, the impact of this potential bias could not be evaluated. Therefore, the follow-up cohort may not have been fully representative of the underlying HPV-OPC patient population, limiting external validity and generalizability. Estimates of PPV/NPV are further influenced by the prevalence of disease recurrence in the study population and may not generalize to different populations, as treatment outcomes may vary between different geographical locations due to various factors, including differing standards of care. Longitudinal sampling was scheduled at regular intervals, yet the restrictions imposed during the pandemic also led to varied sampling timing. Additional studies with more participants covering a higher number of recurrences and longer follow-up are needed to confirm our findings, as the performance metrics could not be precisely determined due to a small number of recurrences. Of note, our reported metrics should be interpreted as measures of agreement between HPV cfDNA results and the biopsy-based clinical reference due to the lack of definition of a perfect gold standard for recurrence detection. Furthermore, studies with larger patient cohorts are required to determine whether patient or tumor characteristics, such as disease stage, influence test performance. Misclassification of tumor HPV status could not be completely ruled out, as the classification algorithm was largely dependent on p16 IHC, which has been shown to lack specificity^9^. Lastly, it remains unclear whether incorporating HPV cfDNA testing into follow-up protocols for HPV-OPC patients adds clinical value. Therefore, data from studies such as this one could be used as a basis to consider how it may be included in clinical decision-making. The benefits would need to be thoroughly assessed in clinical trials before a decision on a potential incorporation into the standard of care can be made.

A strength of this study consisted of the close patient monitoring with a higher frequency of follow-ups than routine aftercare. Analysis of the numerous blood samples, especially those collected closely after diagnosis and throughout treatment, provided insight into HPV cfDNA kinetics during treatment. Tumor HPV status was determined using three established biomarkers, combining p16 IHC with HPV DNA and HPV E6 antibodies to mitigate limitations of a single biomarker. Moreover, the dPCR assay covered a total of eight high-risk HPV types in addition to beta-globin.

In conclusion, our study has demonstrated that HPV cfDNA may in some cases detect recurrence of HPV-OPC multiple months in advance compared to current follow-up protocols. It adds evidence that HPV cfDNA may be a monitoring biomarker for HPV-OPC, even though larger studies are necessary to accurately determine the performance metrics. Early and minimally invasive detection of disease recurrence through complementary use of HPV cfDNA may lead to more effective use of resources during follow-up examinations. Evaluating its clinical value in terms of potentially improved patient outcomes requires clinical trials that compare follow-up protocols incorporating HPV cfDNA testing with the current standard of care.

## Supplementary Information

Below is the link to the electronic supplementary material.

Supplementary Material 1

## Data Availability

Raw data for this study were generated at DKFZ Heidelberg. Data are available upon reasonable request.
