# Supplementary material for: Detection of recurrence of HPV-driven oropharyngeal cancer by HPV cell-free DNA
Source: Sci Rep. 2026 Jun 13;16:18349. doi: 10.1038/s41598-026-56914-7 (PMC13263334; doi:10.1038/s41598-026-56914-7)
Supplement: Supplementary file 1 — Supplementary Material 1 [file 41598_2026_56914_MOESM1_ESM.docx]

**Digital PCR assay**

Supplementary Table 1: Primer and probe details

| target | forward primer | probe | reverse primer |
| --- | --- | --- | --- |
| HPV16 | CAAGTGTGACTCTACGCTTCG | FAM-TGCGTACAAAGCACACACGTAGACA-BHQ1 | GCCCATTAACAGGTCTTCC |
| HPV33 | TTTAYATCCTGAACCAAYTGACC | HEX-CTGCTATGAGCAATTADGTGACAGCTCAG-BHQ1 | GTCCAAGCCTTCATCCTC |
| HPV52 | GCAACCTGAAACAACTGACC | HEX-CTGCTATGAGCAATTADGTGACAGCTCAG-BHQ1 | CACACCATCTGTATCCTCCTC |
| HPV58 | TTTAYATCCTGAACCAAYTGACC | HEX-CTGCTATGAGCAATTADGTGACAGCTCAG-BHQ1 | GTCCAAGCCTATTTCATCCTC |
| HPV18 | TGAAATTCCGGTTGACC | TEX-ACGAGCAATTAAGCGASTCAGAGGA-BHQ2 | GATGATTAACTCCATCTATTTCATCG |
| HPV45 | ATTRGATCCTGTTGACCTGTTG | TEX-ACGAGCAATTAAGCGASTCAGAGGA-BHQ2 | CCATCTGCTTCATCGTTTTC |
| BG | GCCCATCACTTTGGCAA | Cy5-TGGCTAATGCCCTGGCCC-BBQ | AGAAAGCGAGCTTAGTGATAC |
| HPV31 | TTGGAATCGTGTGCCC | Cy5.5-CTACAATGGCTGATCCWGCAGGTACAGAT-BBQ | ATCCCGTCCCCTCCC |
| HPV35 | CCGGCTGTTCACAGAGAG | Cy5.5-CTACAATGGCTGATCCWGCAGGTACAGAT-BBQ | ATCCCGTCCCCTCCC |

Supplementary Table 2: dPCR cycling conditions

| Temperature | Duration [mm:ss] | Cycles |
| --- | --- | --- |
| 95 °C | 02:00 |  |
| 95°C | 00:15 | x 40 |
| 60 °C | 00:30 |  |


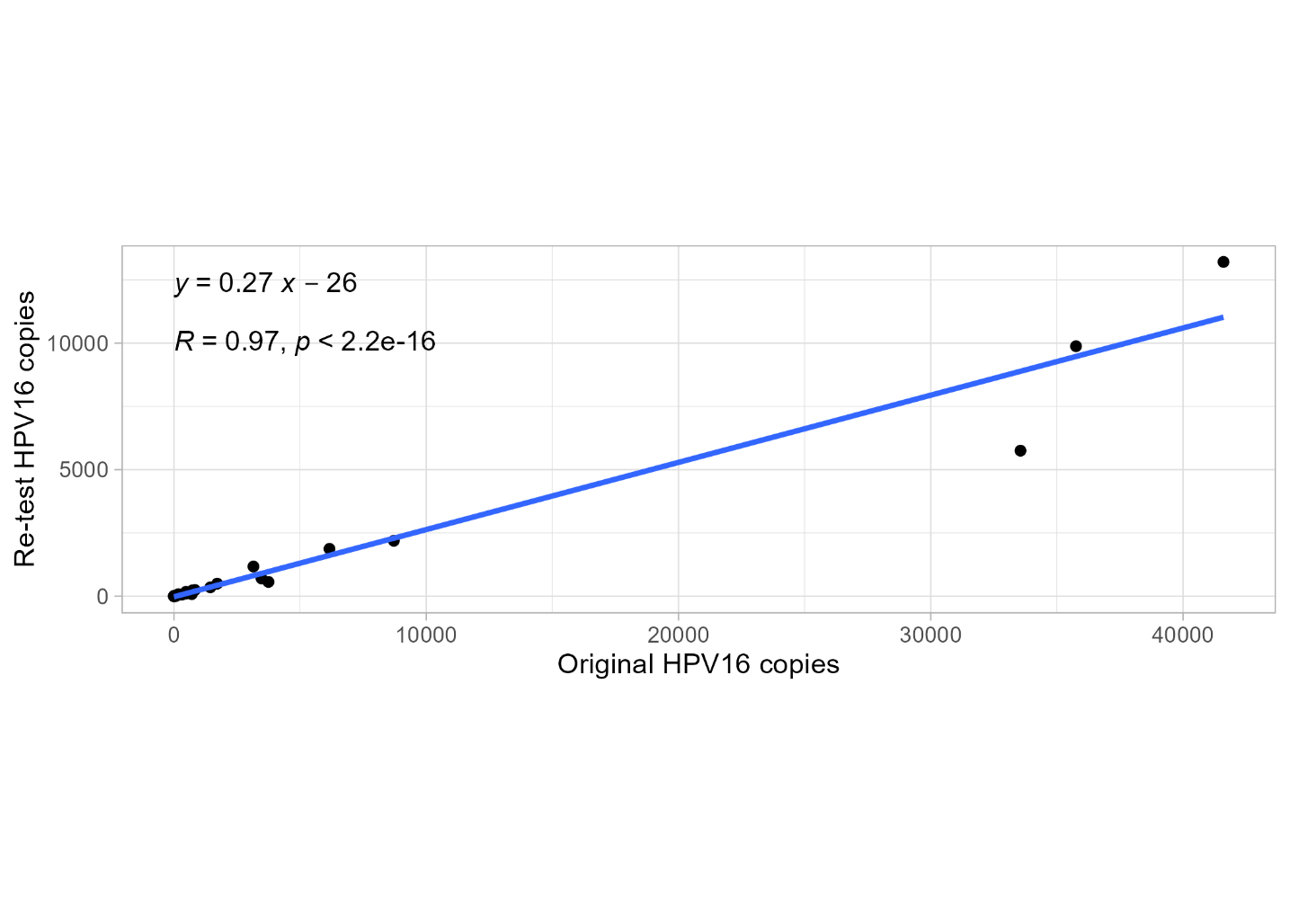


Supplementary Figure 1: Comparison of HPV16 dPCR results between first test and re-test (when HPV31/HPV35-probe was added). Each dot represents an HPV16-positive sample. As expected, reduced number of HPV16 copies were found in the re-test in accordance with the reduced input volume (5 µl vs. 20 µl).

**Sensitivity analysis**

Supplementary Table 3: Sensitivity analysis showing the change in performance metrics if inconclusive test results are classified as positive or excluded from analysis instead of being classified as negative. Each sample was tested in dPCR duplicates. If a positive partition was present in only one of the duplicates, the result was labeled as inconclusive. In our primary/final analysis, inconclusive test results were treated as negative, as specificity was prioritized. Square brackets indicate the 95% Clopper-Pearson confidence interval.

|  | **Primary analysis: inconclusive classified as negative** | **Alternative 1: inconclusive classified as positive** | **Alternative 2: inconclusive excluded** |
| --- | --- | --- | --- |
| **Sensitivity at diagnosis** | 95% (36/38) [82-99%] | 97% (37/38) [86-100%] | 97% (36/37) [86-100%] |
| **Specificity at diagnosis** | 95% (19/20) [75-100%] | 80% (16/20) [56-94%] | 94% (17/18) [73-100%] |
| **Sensitivity post-treatment** | 33% (3/9) [7-70%] | 56% (5/9) [21-86%] | 50% (3/6) [12-88%] |
| **Specificity post-treatment** | 98% (49/50) [89-100%] | 88% (44/50) [76-95%] | 98% (44/45) [88-100%] |
| **Positive predictive value (PPV)** | 75% (3/4) [19-99%] | 45% (5/11) [17-77%] | 75% (3/4) [19-99%] |
| **Negative predictive value (NPV)** | 89% (49/55) [78-96%] | 92% (44/48) [80-98%] | 94% (44/47) [82-99%] |

**Follow-up overview**

Supplementary Table 4: Overview table for 24 HPV-OPC patients with follow-up samples.

| **Patient ID** | **Total number of samples** | **Number of samples tested positive for HPV cfDNA** | **Number of samples tested negative or inconclusive for HPV cfDNA** | **Outcome: OPC recurrence or persistence within follow-up period (yes/no)** | **Follow-up period (in days)** |
| --- | --- | --- | --- | --- | --- |
| **1** | 4 | 3 | 1 | yes | 658 |
| **2** | 9 | 5 | 4 | yes | 610 |
| **3** | 4 | 2 | 2 | yes | 352 |
| **4** | 4 | 1 | 3 | yes | 422 |
| **5** | 3 | 1 | 2 | no | 261 |
| **6** | 3 | 3 | 0 | no | 254 |
| **7** | 2 | 2 | 0 | no | 254 |
| **8** | 8 | 1 | 7 | no | 868 |
| **9** | 9 | 3 | 6 | no | 721 |
| **10** | 8 | 1 | 7 | no | 700 |
| **11** | 7 | 2 | 5 | no | 651 |
| **12** | 7 | 1 | 6 | no | 672 |
| **13** | 7 | 3 | 4 | no | 713 |
| **14** | 7 | 1 | 6 | no | 681 |
| **15** | 6 | 1 | 5 | no | 609 |
| **16** | 5 | 1 | 4 | no | 595 |
| **17** | 3 | 1 | 2 | no | 649 |
| **18** | 2 | 1 | 1 | no | 469 |
| **19** | 3 | 1 | 2 | no | 464 |
| **20** | 3 | 2 | 1 | no | 442 |
| **21** | 3 | 2 | 1 | no | 436 |
| **22** | 3 | 2 | 1 | no | 410 |
| **23** | 2 | 1 | 1 | no | 352 |
| **24** | 3 | 2 | 1 | no | 254 |
